# The Effect of High Concentrations of Glufosinate Ammonium on the Yield Components of Transgenic Spring Wheat (*Triticum aestivum* L.) Constitutively Expressing the *bar* Gene

**DOI:** 10.1100/2012/657945

**Published:** 2012-05-02

**Authors:** Zoltán Áy, Róbert Mihály, Mátyás Cserháti, Éva Kótai, János Pauk

**Affiliations:** ^1^Department of Biotechnology, Cereal Research Non-Profit Ltd. Co., Alsó kikötő sor 9, 6726 Szeged, Hungary; ^2^Biological Research Centre, Institute of Plant Biology, Hungarian Academy of Sciences, Temesvári körút 62, 6726 Szeged, Hungary

## Abstract

We present an experiment done on a *bar* ^+^ wheat line treated with 14 different concentrations of glufosinate ammonium—an effective component of nonselective herbicides—during seed germination in a closed experimental system. Yield components as number of spikes per plant, number of grains per spike, thousand kernel weight, and yield per plant were thoroughly analysed and statistically evaluated after harvesting. We found that a concentration of glufosinate ammonium 5000 times the lethal dose was not enough to inhibit the germination of transgenic plants expressing the *bar* gene. Extremely high concentrations of glufosinate ammonium caused a bushy phenotype, significantly lower numbers of grains per spike, and thousand kernel weights. Concerning the productivity, we observed that concentrations of glufosinate ammonium 64 times the lethal dose did not lead to yield depression. Our results draw attention to the possibilities implied in the transgenic approaches.

## 1. Introduction

Effective weed control has become one of the most significant procedures in cropping operations to ensure good quality harvests. Due to the high costs of energy required, mechanical weed control practices are now viewed as unsatisfactory and have been largely replaced by chemical weed control using herbicides. Herbicides generally function by disrupting unique and essential processes in plants, for example, photosynthesis, pigment biosynthesis, mitosis, or essential amino acid biosynthesis [1].

Amino acid biosynthesis is one of the pathways targeted most by herbicides. The discovery of a peptide antibiotic produced by the actinomycetes *Streptomyces viridochromogenes *and *S. hygroscopicus *was reported several decades ago [2, 3]. The antibiotic, named PTT (phosphinothricin-tripeptide = phosphinothricyl-alanyl-alanine = bialaphos), consists of two molecules of L-alanine and one molecule of the rare amino acid PT (L-phosphinothricin). According to the postulated biosynthetic pathway, PT is generated from two molecules of phosphoenolpyruvate, one molecule of acetyl coenzyme A and one methyl group of methylcobalamin in thirteen biosynthetic steps [4, 5]. The bioactive component of the PTT molecule is the PT which, as a structural analogue of glutamic acid, interferes with amino acid synthesis through the competitive, irreversible inhibition of GS (glutamine synthetase), the key enzyme of nitrogen metabolism [6, 7]. The inhibition of GS reduces glutamine acid levels and triggers ammonium ion accumulation to levels up to 100-fold higher than in control cells [8, 9]. Due to this, PT has bactericidal, fungicidal, and herbicidal properties. In the case of plants, two to four hours after application of PT, photosynthesis slows down and plants yellow and die in two to five days [10].

Since many herbicides are nonselective, both crops and weeds share the processes mentioned above. For instance, over 40 monocotyledonous and more than 150 dicotyledonous species are sensitive to PT [11]. Consequently, selectivity must be based on the different ways herbicides act upon weeds and crops. The most effective approach to achieve this goal is the development of crop cultivars with tolerance to the so-called broad-spectrum herbicides by using plant biotechnology techniques such as *in vitro *cell culture, mutagenesis, or genetic transformation followed by selection under herbicide pressure. Tolerance via genetic transformation can be conferred by modification of the herbicide target enzyme in such a way that the herbicide molecule does not bind to the target enzyme or introduction of a gene coding for a herbicide detoxifying enzyme [1, 12].

Usually, genes coding for proteins useful in herbicide resistance in crops can be isolated from herbicide degrading soil microorganisms. The strategy to develop PT resistant crops is based on the mechanism used by PTT-producing actinomycetes, which can protect themselves against the autotoxic effect. This pathway is mediated by the enzyme PAT (phosphinothricin-N-acetyltransferase) which acetylates the free amino group of PT, thereby causing its detoxification. The PAT-encoding *bar *(bialaphos resistance) and *pat *genes were isolated from *Streptomyces hygroscopicus *[13–15] and *S. viridochromogenes *Tü494 [16], respectively. Both genes code for PAT proteins of 183 amino acids, which show 85% homology to each other, variations of the genes being confined to their noncoding regions [17].

Glufosinate ammonium is a proherbicide which is converted by plant cells into PT. Originally it was engineered by Hoechst in the 1970s for preharvest desiccation in potato, legumes, and oilseed rape. Since the discovery of the *bar/pat* gene system, glufosinate ammonium has found its applications in weed control and in selection of transgenic plants expressing resistance genes. It is marketed under a number of trade names including Basta, Challenge, Finale, and Radicale. Engineering tolerance to glufosinate ammonium in crops including wheat by genetic modification has been studied by many research groups [18–22].

The present study is the first which describes an experiment with a transgenic line of spring wheat constitutively expressing the gene *bar *in order to determine the extent of herbicide resistance and the complex effect of extremely high concentrated glufosinate ammonium on different yield parameters.

## 2. Materials and Methods

### 2.1. Genetic Transformation and Selection of Transgenic Plants

Spring wheat plants (*Triticum aestivum*, L., cv. CY-45) were grown in the greenhouse. Donor spikes were harvested 12–14 days after flowering. Embryos were excised from surface-sterilized immature seeds and plated onto callus induction medium. Gene transfer via particle bombardment was carried out according to Altpeter et al. [23]. The vector pAHC25 [24] containing the gene *bar* regulated by a constitutive maize ubiquitin promoter was used for genetic transformation. Putative transgenic plantlets were transferred to the soil in the greenhouse after a 4–6-week period of *in vitro *regeneration. After molecular studies, plants were sprayed with the wide-range herbicide Finale 14 SL (IUPAC name: *methyl(E)-methoximino-*{*(E)-a-[1-(a,a,a-trifluoro-m-tolyl)ethylide-neaminooxy]-o-tolyl*}*-acetate*; active ingredient: 150 g · L^−1^ glufosinate ammonium) at 1.0% v/v, as recommended by the manufacturer. Survivor plants were grown and harvested. Progenies were also grown in the greenhouse alike and self-pollinated through six generations in order to acquire homozygous wheat lines, thereby eliminating the possibility of the segregation of the *bar* gene. Nontransgenic individuals were selected according to the results of molecular genetic methods and were eliminated by being sprayed with Finale 14 SL solution in every generation.

### 2.2. Test for Herbicide Resistance

As a benchmark, the lethal dose of glufosinate ammonium was defined in a preliminary experiment. Mature embryos were excised from surface-sterilized seeds of the nontransgenic spring wheat variety CY-45 and were *in vitro* germinated in tubes, containing 5 mL of half-strength MS_0_ medium [25] supplemented with 0, 1, 2, and 4 mg · L^−1^ of glufosinate ammonium (C_5_H_15_N_2_O_4_P; 198.16 g/mol; Sigma), respectively. Incubation was carried out in a growing chamber (24°C, 16 h light/8 h dark photoperiod) and results were evaluated on the tenth day of culture.

The resistance test was carried out with the transgenic spring wheat line “T-124” in the seventh self-pollinated generation (T_7_). The gene *bar* had one integration site in this wheat line. Culture conditions during germination of the mature embryos were the same as in the pilot experiment. Media representing fourteen treatments with different concentrations of glufosinate ammonium added to them were as follows: 2, 4, 8, 16, 32, 64, 128, 200, 400, 600, 800, 1000 and 5000 mg · L^−1^. Medium of the control treatment contained no herbicide. One embryo was put into every tube and every treatment was repeated eight times. After three weeks of culture, plantlets were transferred to pots filled with soil, acclimatized and grown to maturity in the greenhouse. Plants were sprayed with insecticides and fungicides twice during the growing period. Exclusively mechanical weed control was also applied. Spikes were harvested individually and sorted into two groups termed well filled and low filled according to visual qualification. Yield components as number of spikes per plant, number of grains per spike, and yield per plant were measured while thousand kernel weight was calculated after harvesting.

### 2.3. Molecular Assays

Plantlets were analyzed by molecular methods in every transgenic generation. At the seedling stage, 30 mg of leaf samples were collected and immediately frozen in liquid nitrogen. For the purification of total RNA, the “SV Total RNA Isolation System” kit (Promega) was applied; the protocol also contained the DNase treatment. To prove not only the presence but also the expression of the *bar* gene, a fragment 375 bp in length derived from its RNA transcript was amplified by RT-PCR (one step reverse transcriptase polymerase chain reaction) with the aid of the specific primers bar5F and bar6R (5′-CAGGAACCGCAGGAGTGGA-3′ and 5′-CCAGAAACCCACGTCATG-3′, resp.). RT-PCR products were detected by electrophoresis on 1% TAE-agarose gel. Only the *bar* ^+^ plants were grown to maturity and harvested in every generation. Concerning the resistance test population, one out of the eight individuals was randomly chosen in each herbicide treatment and analyzed as described above.

### 2.4. Experimental Conditions of Transgenic Research

Transgenic experiments were carried out in closed experimental conditions (*in vitro* growing chamber and closed greenhouse cabin). After the observations destruction of experimental plant material was documented in an official report for the Hungarian authorities.

### 2.5. Statistical Evaluation

Results of well-filled and low-filled groups were evaluated separately. In every treatment, main rates were calculated by averaging of the results of the eight repeats. Data of partially and totally sterile spikes were also included in the statistical analysis using Microsoft Excel 2003 software (Microsoft Inc., USA). The effect of glufosinate ammonium on the agronomical parameters was evaluated by one-way analysis of variance (one-way ANOVA).

## 3. Results

**Figure 1:**
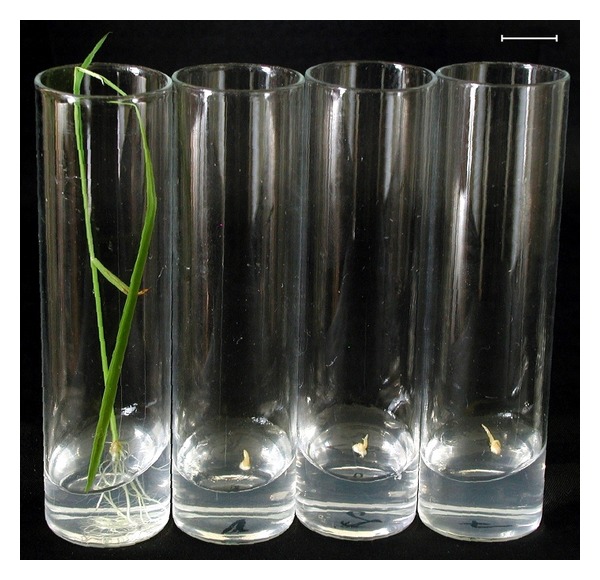
Germination of mature embryos of the nontransgenic spring wheat variety CY-45 on media containing 0, 1, 2, and 4 mg · L^−1^ glufosinate ammonium (from left to right) on the tenth day of culture. bar * 1.0 cm.

In a preliminary experiment, we defined the lethal dose of glufosinate ammonium. Embryos excised from the nontransgenic spring wheat variety CY-45 were germinated *in vitro*. During each repeat experiment, only those embryos germinated which were placed onto medium without any glufosinate ammonium while 1–4 mg · L^−1^ effective medium concentration resulted in neither shoots nor roots (Figure 1). This information revealed that, during germination, the lethal dose of glufosinate ammonium must be less than 1 mg · L^−1^ in this experiment.

**Figure 2:**
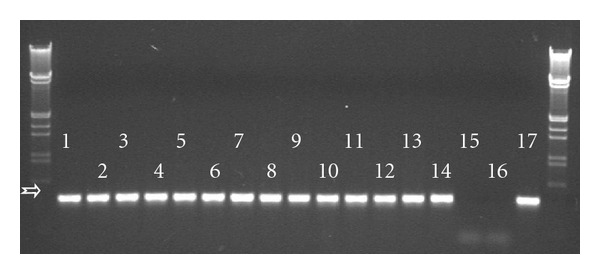
Detection of RNA transcripts derived from the herbicide resistance gene *bar* by electrophoresis of RT-PCR products. The white arrow shows the expected 375 bp fragment. Markers: *λ*-DNA digested with EcoRI and HindIII restriction enzymes. Samples from left to right: 1–14: according to increasing herbicide concentrations (1 refers to 0 while 14 refers to 5000 mg · L^−1^ of glufosinate ammonium), 15: nontransgenic CY-45 plant, 16: distilled water, 17: pAHC25 plasmid DNA.

**Figure 3:**
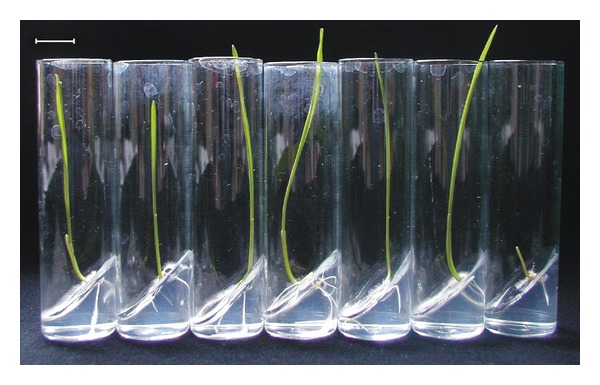
Germination of mature embryos of the transgenic spring wheat line “T-124” on media containing 0, 200, 400, 600, 800, 1000, and 5000 mg · L^−1^ glufosinate ammonium (from left to right) on the seventh day of culture. bar * 1.0 cm.

**Figure 4:**
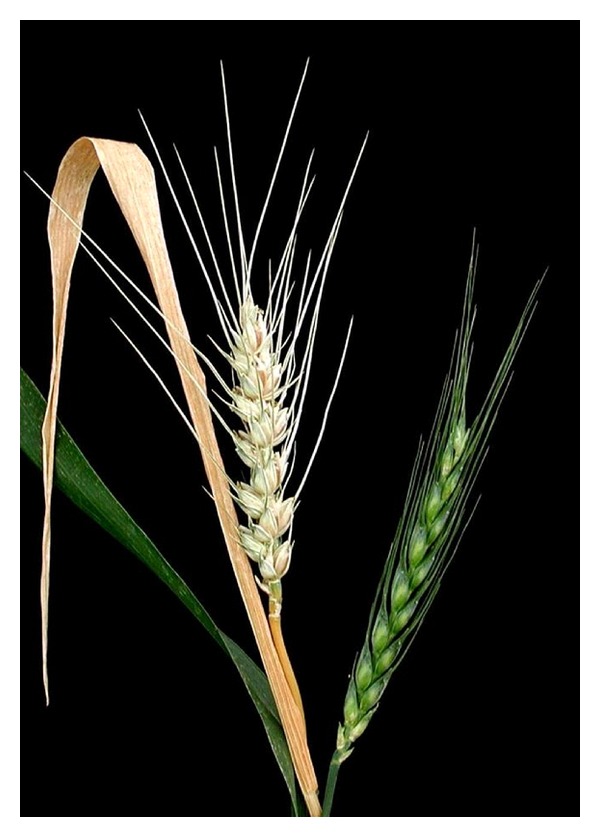
Plants treated with 5000 mg · L^−1^ glufosinate ammonium during germination (on the right) had growing period three weeks longer than the untreated control ones (on the left).

**Figure 5:**
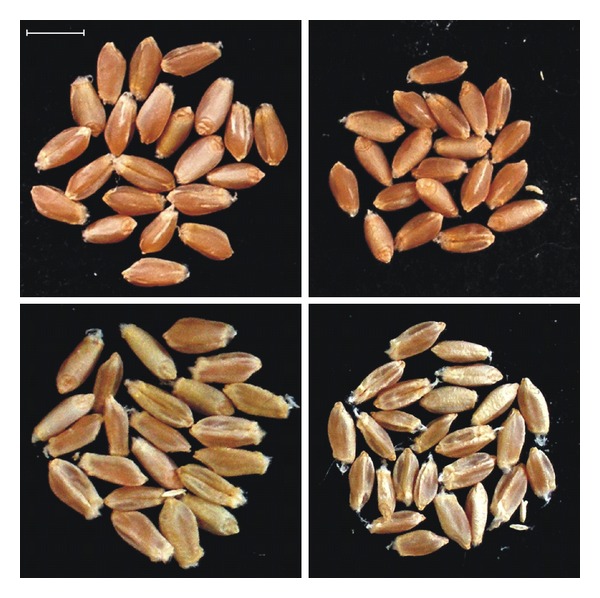
Grains of the well-filled (on the top) and the low-filled spikes (on the bottom). Control (on the left) and 5000 mg · L^−1^ (on the right) treatments resulted in different size and exterior of grains. bar * 0.5 cm.

In the course of the test for herbicide resistance of the transgenic wheat line “T-124,” as it was expected, genetic segregation of the *bar* gene was not observed in the experimental plant population. This fact was confirmed by RT-PCR as well (Figure 2). Every embryo germinated under herbicide pressure; consequently, the resistance test was done with 112 transgenic wheat plants. Embryos germinated with the same intensity but, noticeably, the presence of 5000 mg · L^−1^ glufosinate ammonium in the medium led to slower germination. Plantlets had shoots only 1 cm in length on the seventh day of culture while those growing on the other media had shoots 11-12 cm in length at the same timepoint (Figure 3). Those treated with 5000 mg · L^−1^ glufosinate ammonium during germination stayed behind in development and growth compared to the others throughout the entire growing period. They only began to flower when the others had already been ready for harvesting (Figure 4), and finally, their growing period was prolonged by three weeks. In spite of these observations, every plantlet grew to maturity and developed 773 spikes in total (100%). According to visual qualification of the seeds, 311 spikes (40.2%) were considered as well filled while 462 others (59.8%) proved to be low filled (Figure 5). Obviously, partial and total sterility occurred only among the low-filled ones (19 spikes (2.4%) and 7 spikes (0.9%), resp.).

**Figure 6:**
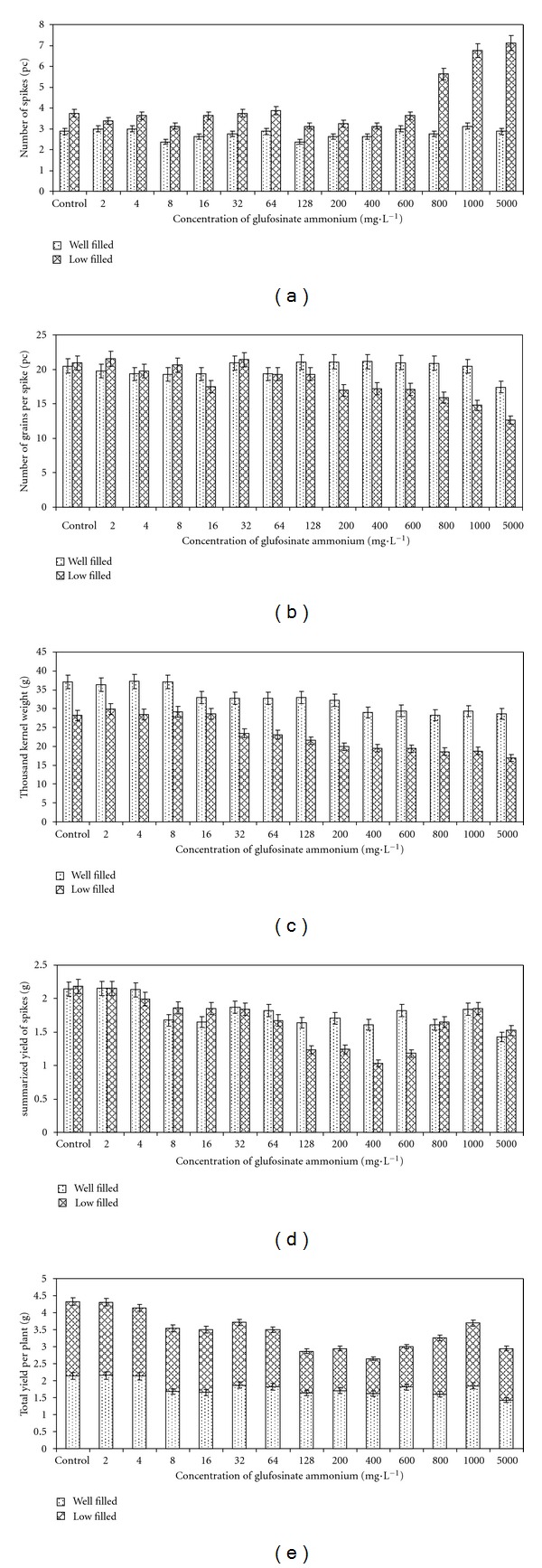
Effect of different concentrations of the herbicide glufosinate ammonium on the number of spikes (a), number of grains per spike (b), thousand kernel weight (c), summarized yield of spikes (d), and total yield per plant (e) of the transgenic wheat line “T-124.” Values are equal to the average of the eight repeat experiments.

The number of spikes per plant varied between 2.375 and 3.125 in the well-filled group. These data represent the same level of significance (Table 1). By contrast, this parameter was similar in the case of low-filled spikes but strongly increased at the three highest concentrations of glufosinate ammonium. Plants treated with 5000 mg · L^−1^ herbicide showed the most intensive shoot development (Figure 6(a)) causing a bushy phenotype. Data in this group corresponded to three levels of significance (Table 1). 

The highest value of the number of grains per well-filled spikes was 21.1 while the lowest was 17.4. The latter one was a result of application of 5000 mg · L^−1^ glufosinate ammonium and it is significantly lower than the other values (Table 1). Compared to this, the number of grains per spike was lower in the low-filled group and varied between 21.4 and 12.6 (Figure 6(b)). These data correspond to three levels of significance. Interestingly, 16 and 200–800 mg · L^−1^ of glufosinate ammonium resulted in the same level of significance (Table 1).

The thousand kernel weight was calculated after the yield of the spikes was harvested. Obviously, drastic differences were found between the two main groups. Representing three levels of significance (Table 1), weight values of the well-filled spikes varied from 37.1 g to 28.6 g. Contrary to this, data of the low-filled spikes indicated four levels (Table 1) where the weight value changed between 29.8 g and 16.9 g (Figure 6(c)).

Yield per spikes was summarized before evaluation both in well-filled and low-filled groups in order to receive the yield per plant. This parameter showed similarity between the two groups since values in the well-filled group varied from 2.15 g to 1.42 g and in the other case from 2.18 g to 1.03 g (Figure 6(d)). There were no significant differences between the well-filled spike groups (Table 1) but, noticeably, a significantly lower yield in the low-filled group was due not to treatments with the highest concentration of glufosinate ammonium but rather to treatments with a concentration of only 128–600 mg · L^−1^ (Table 1).

Since yield is the most important agronomical parameter, we also represent the total yield per plant by summarizing the results of the well-filled and the low-filled groups. In this case, data varied between 4.32 g and 2.64 g (Figure 6(e)). Compared to the control plants, total yield of those treated with 128–5000 mg · L^−1^ glufosinate ammonium—except the 1000 mg · L^−1^ one—significantly decreased below 3 grams (Table 1).

**Figure 7:**
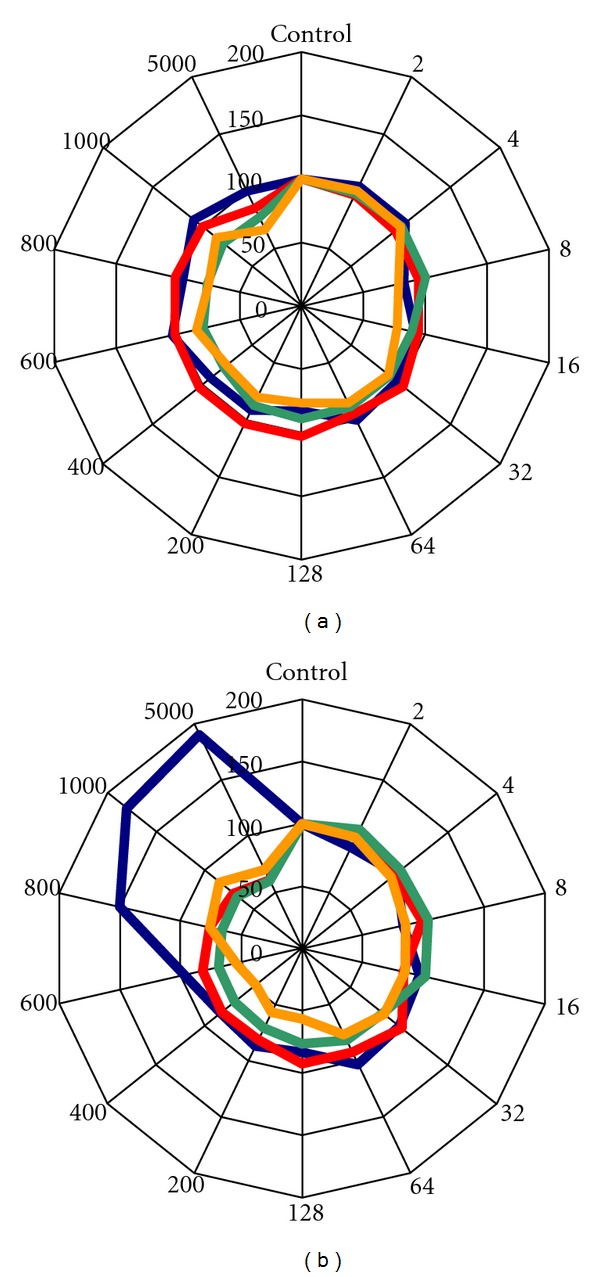
Complex effect of glufosinate ammonium on the well-filled (a) and the low-filled spikes (b). Colour key: blue—number of spikes; red—number of grains per spikes; green—thousand kernel weight; yellow—summarized yield of spikes. Control treatment represents 100 per cent. Values are equal to the average of the eight repeat experiments.

To form a more detailed picture of the complex effect of glufosinate ammonium on the yield components, we represent the results also in cycle diagrams (Figure 7). The most conspicuous divergence between the well-filled and low-filled groups was the increase in the number of spikes up to 190% under extremely high concentration of the herbicide. Other parameters showed similar changes but not similar significance levels, showing that the yield parameters changed the same way in both well-filled and low-filled groups.

## 4. Discussion

Initial growth conditions play a key role in the life cycle of a plant and they determine the vigour during the seedling stage. According to our former observations, wheat was the most sensitive to PT-like herbicides exactly during seed germination (data not shown). Therefore, we exposed transgenic wheat embryos to different concentrations of the herbicide glufosinate ammonium which is converted by plant cells into PT.

In the preliminary experiment, we found that less than 1 mg · L^−1^ of glufosinate ammonium in the culture medium is enough to inhibit CY-45 (wild-type) embryo germination. Similarly low concentrations of PT-like herbicides made possible the successful selection of transgenic tissues according to pioneer wheat transformation studies [26–28]. 

Throughout the first three weeks of their life cycle, wheat plantlets derived from the transgenic plant line “T-124” constitutively expressing the *bar *gene were challenged by 14 different concentrations of glufosinate ammonium. By transferring the plants into the soil, herbicide pressure was stopped and every plantlet was grown to maturity under the same conditions. Nevertheless, those treated with higher concentrations of glufosinate ammonium showed significant differences in the four examined parameters compared to the controls, thus, these divergences were clearly the aftermath of the herbicide treatment and confirm the importance of growth conditions during seed germination.

Our purpose was to determine the extent of herbicide resistance and the complex effect of extremely high concentrations of glufosinate ammonium. Therefore, we evaluated the application of the herbicide not with the well-known scale method but with exact and repeatable measurement of yield parameters such as the number of spikes per plant, grains per spike, thousand kernel weight, and yield per plant as an objective standard. Besides these important traits, we also recorded the length of the growing period of the plants which had been prolonged strikingly by the influence of the highest concentration (5000 mg · L^−1^) of the herbicide. It is probable that so many glufosinate ammonium molecules were converted into PT molecules in the cells that in spite of the constitutive production of the PAT enzyme, plants could detoxify the herbicide only at the expense of slowed down metabolism which led to the absorption of fewer nutrients from the culture medium. Plants tried to compensate for this lag after the transfer into soil made manifest not in the strengthening of the main shoot but in developing several lateral shoots. Those individual plants treated with 800 and 1000 mg · L^−1^ of glufosinate ammonium showed a similar stool phenotype at harvest time which suggests that all the three highest concentrations of herbicide targeted the plants seriously. Certain studies reported that PT applied in levels lower than the lethal dose stimulates *in vitro* shoot regeneration in the case of grape [29], snapdragon [30], and rice [31, 32]. Our results reveal that increased ammonium ion level within the plant cell might act as a source of abiotic stress. Therefore, according to the apical dominance theory, inhibition of the apical tissues can lead to more intensive lateral shoot growth. However, this kind of escape was coupled with a weaker condition, which developed low-filled spikes without exception. 

The reason why we sorted the spikes into well-filled and low-filled groups was to get a more detailed picture of the complex effect of glufosinate ammonium on the yield components. Table 1 shows the differences between these groups very well. The decrease in the number of grains per spike was caused mainly by the shortening of spikes but in the case of 16, 200 and 5000 mg · L^−1^ treatments, this was supplemented with partial or total sterility of low-filled spikes (data not shown). Thousand kernel weight decreased with almost the same intensity in both groups. However, changes in the values of this index did not manifest themselves in the summarized yield of spikes in the well-filled group because the stable number of spikes and number of grains per spike offset them. Quite different phenomena were observed in the low-filled group. Lower thousand kernel weights began to cause a decrease in the summarized yield of spikes at 128 mg · L^−1^ of glufosinate ammonium, but this tendency was reversed at 800 mg · L^−1^ and higher concentrations. This decrease can be traced back unambiguously to the negative changes in thousand kernel weight and number of grains per spike while the increase was caused by the higher number of spikes. Total yield per plants fluctuated similarly but only the 1000 mg · L^−1^ treatment could reverse the reduction. We did not check the quality of the grains in this experiment but we must make it absolutely clear that in the case of the three highest concentrations of glufosinate ammonium, the yield was restored definitely by the increased number of low-filled spikes representing a visibly poor quality. Briefly, plants could compensate the effect of extremely high concentrations of herbicide only at the expense of tissue deterioration, which is a kind of yield depression.

To make the above results comparable with other studies, we consider writing a study on the importance of the extent of resistance in plants, all the more so, since similar publications describe the sensitivity of plants to herbicides in different ways. If we take plant death as a basis we cannot say by how much more resistant our transgenic plants are compared to the control ones since all of them survived the 5000 mg · L^−1^ treatment, thus the lethal dose remained unknown. If we take the slightest significant change in the examined parameters we can see that the 8 mg · L^−1^ treatment was the highest which caused no significant difference. We think that both of these approaches are misleading; therefore we chose the total yield, the most important trait of cereal crops, as a benchmark. We found that 64 mg · L^−1^ was the highest concentration of herbicide which caused no significant loss in the yield. Consequently, a threshold value of resistance to glufosinate ammonium must be between 64 and 128 mg · L^−1^ according to this experiment. Since the lethal dose of the herbicide proved to be less than 1 mg · L^−1^, transgenic plants therefore achieved at least 64-fold resistance. This value is undoubtedly higher than those published in articles not only about PT [11, 31, 33–35] but also about other types of herbicides like imidazolinones [36] and glyphosate [37–40].

We suggest that this kind of high herbicide resistance should not be utilized in practice because it can lead to the rapid natural development of resistant weed populations [41]. We set a rather high theoretical value on our results as researchers will need to analyze the impacts of many transgenes with similar rigour in the near future.

## Figures and Tables

**Table 1:** Significance levels of the averages of the eight repeat experiments at LSD_005_ in the well-filled (i) and in the low-filled (ii) groups and in case of the total yield per plants (iii) according to one-way analysis of variance (one-way ANOVA).

(i)	LSD_5%_	cont.	2	4	8	16	32	64	128	200	400	600	800	1000	5000
Spikes per plant (pc)	1,051	a	a	a	a	a	a	a	a	a	a	a	a	a	a
Grains per spikes (pc)	1,893	a	a	a	a	a	a	a	a	a	a	a	a	a	b
Thousand kernel weight (g)	1,855	a	a	a	a	b	b	b	b	b	c	c	c	c	c
Sum. yield of spikes (g)	0,836	a	a	a	a	a	a	a	a	a	a	a	a	a	a

(ii)	LSD_5%_	cont.	2	4	8	16	32	64	128	200	400	600	800	1000	5000

Spikes per plant (pc)	1,111	a	a	a	a	a	a	a	a	a	a	a	B	C	C
Grains per spikes (pc)	2,274	a	a	a	a	b	a	a	a	b	b	b	b	c	c
Thousand kernel weight (g)	1,947	a	a	a	a	a	b	b	b	c	c	c	c	c	d
Sum. yield of spikes (g)	0,734	a	a	a	a	a	a	a	b	b	b	b	a	a	a

(iii)	LSD_5%_	cont.	2	4	8	16	32	64	128	200	400	600	800	1000	5000

Total yield of plants (g)	0,846	a	a	a	a	a	a	a	b	b	b	b	b	a	b
